# The folk illness *kimeo* and “traditional” uvulectomy: an ethnomedical study of care seeking for children with cough and weakness in Dar es Salaam

**DOI:** 10.1186/s13002-022-00533-9

**Published:** 2022-04-29

**Authors:** Siri Lange, Dorcas Mfaume

**Affiliations:** 1 Department of Health Promotion and Development (HEMIL), University of Bergen, P.O. Box 7800, 5020 Bergen, Norway; 2 Dar es Salaam, Tanzania

## Abstract

**Background:**

Amputation of the uvula by lay providers, so-called “traditional uvulectomy”, is common in parts of Sub-Saharan Africa. In Tanzania, the procedure is a treatment of persistent cough, and in some areas of the country, one in three children have been cut. Previous research from Sub-Saharan Africa suggest that uvulectomy by lay providers can increase morbidity and mortality in children, but few studies have examined the cultural ideas and practices that are linked to this form of lay surgery.

**Methods:**

This ethnomedical study took place in Dar es Salaam. Ten focus group discussions with a total of 43 caregivers in three different administrative districts were carried out, focusing on their perceptions of uvulectomy, the folk illness *kimeo,* and their experiences with taking a child for cutting. Four folk practitioners who carry out uvulectomies were interviewed individually, with a special focus on their background, and their perceptions of *kimeo* and uvulectomy.

**Results:**

Caregivers in Dar es Salaam typically take children who suffer from cough, vomiting and weakness to a professional health provider as a first recourse. If the child does not get well relatively quickly, some start fearing that their child may suffer from the folk illness *kimeo*. *Kimeo* is perceived by some to be an acute, life-threatening illness that professional health providers are incapable of treating. Folk practitioners treat *kimeo* by amputating the uvula using forceps. The four interviewed practitioners had learned their skill in apprenticeship, and two of them were third generation uvula cutters. Caregivers regard the folk practitioners as experts who offer a service that is perceived as both efficient and safe.

**Conclusions:**

Efforts should be made to improve the quality of professional health services for children presenting with cough, including more open communication with caregivers about the folk illness *kimeo*. More research is needed to establish the clinical conditions that children who are taken for uvulectomy suffer from, to what degree the practice delays professional health care for underlying illnesses like pneumonia, and the negative effects of the cutting itself.

## Background

Care seeking for children in Sub-Saharan Africa is characterised by medical pluralism, uncertainty, financial obstacles, and poor access to quality services. As a result, caregivers often try out “multiple and simultaneous treatments” when their children are ill [1]. This study focuses on “indigenous” or “traditional” uvulectomy; a surgical practice that has been defined as “an excision of the uvula, usually performed by nonphysician healers” [2]. The practice has been documented in several African countries, including Cameroon, Ethiopia, Nigeria, Niger and Tanzania [2–8]. This form of health service is typically hybrid, and the providers do not necessarily perceive the service that they offer as “traditional”. Nevertheless, the term “traditional uvulectomy” has been established in the public health literature, and most authors refer to the people who conduct the surgery as “healers” or “traditional healers”.

In Dar es Salaam, where this study was conducted, Tanzanians refer to a practitioner who performs this form of surgery as “uvula cutter” (*mkata kimeo,* literally a person who cuts a kimeo), rather than by the Swahili terms that translate to “healer” (*mganga wa kienyeji, mganga wa jadi* or *mtaalam wa tiba asili*). The uvula cutters have learned their skills in apprenticeship. Drawing on Kleinman’s conceptual framework [9], we will use the term “folk practitioner” when we refer to the uvula cutters. Folk practitioners “require training, talents, or experience beyond those available to the population at large” and are “likely to learn as an apprentice” [10]. Folk practitioners can also be described as “non-bureaucratic” and “non-professional” [11]. Professional providers, in contrast, have formal training and certification.

Ethnomedicine studies “notions of health and illness, including how people think and how people act about well-being and healing” in different societies, including in the global North [10]. The term folk illness denotes an illness that is not recognised by biomedical health providers [12]. An ethnomedical study of health-seeking behaviour related to the folk illness *kimeo* can help us understand why caregivers choose to take their children for uvulectomy also in areas with relatively good access to professional health care—in other words the social acceptability of folk practitioners’ practices. The concept of acceptability is multi-faceted, but is linked to the degree to which there is “’fit’ between the clients and the system” [13, 14], or in other words, whether people who receive a given form of healthcare “consider it to be appropriate” [14].

There is often a profound mismatch between professional providers’ and patients/caregivers’ perceptions of appropriate care. In many cases, patients do not perceive folk practitioners’ care as “alternative” or “complementary”, but the only option for appropriate care [4, 15, 16]. Moreover, while discourses in public health tend to conceptualise professional and folk health services as two separate spheres, folk health systems are usually “open systems” [17], ready to integrate foreign or new forms of therapy [18], and folk practitioners are sometimes invited to participate in biomedical projects [19]. Folk providers may use modern medical equipment, selectively prescribe biomedical drugs, or, as documented in both Uganda, Guatemala and Haiti, specialise as folk “injectors” of drugs from pharmacies [10, 20, 21].

### Uvulectomy by professional health staff and folk practitioners

A review of the literature on uvulectomy, published in 1989, found that the procedure was a treatment for tonsillitis in Europe and in the USA in the seventeenth century, but that the practice died out with the introduction of chemotherapy. The review documents widespread uvulectomies in the Middle East and in many countries in Sub-Saharan Africa in the 1980s, but the author predicted that the practice would die out when or if “western” biomedical services would become more available and health education carried out [22]. Research from the last fifteen years, however, shows that the practice is still common in many communities in Sub-Saharan Africa [4, 5, 7, 8]. The surgery encompasses a variety of practices and often varies between different ethnic groups within the same country. In some communities, the cutting has been classified as “ritual” and it is carried out on neonates, while in some it is curative against vomiting and diarrhoea, and in yet others it is a prophylactic measure against vomiting [5, 6, 23, 24].

The practitioners performing uvulectomy normally claim that there are no or limited complications following the procedure [24, 25], but several scholars have argued that the practice can lead to morbidity and mortality, particularly due to haemorrhage and sepsis. These studies look at complications among children who have gone through uvulectomy and who have ended up at hospital [2, 4, 6, 24]. Therefore, we do not know the prevalence of complications among cohorts of children who have gone through uvulectomy. In addition to potential complications from the cutting itself, there is also fear that the practice can contribute to HIV infections, either through unclean instruments or through mother-to-child transmission (MTCT) during lactation by children who have open wounds following uvulectomy [26].

Finally, it has been argued that beliefs associated with uvulectomy can delay the biomedical treatment of illnesses in children, but there are no systematic studies on this aspect. However, a study of twenty malnourished children in Kenya found that for some caregivers, uvulectomy was considered the “normal” treatment for children who are vomiting, and the children were therefore taken for cutting before they were brought to a professional provider [27].

It should be noted that some medical case reports describe rare conditions where children who suffer from cough caused by an abnormally long uvula get better after uvulectomy has been performed [28, 29].

### Uvulectomy in Tanzania

Hospital studies conducted at the national hospital in Dar es Salaam in the 1960s and 1980s found that the prevalence of uvulectomy was very high [30, 31]. One of the studies argues that the belief “in the efficacy of uvulectomy in curing chronic cough appeared to cause considerable delay in the diagnosis of tuberculosis” [30].

Recent studies show that there is great variation in prevalence in different regions in the country. A random sample of children under five in the Coast region found that one in three children under five had undergone uvulectomy. The prevalence among the Zaramo and the Makonde ethnic groups in this sample was particularly high, 75% and 72%, respectively [8]. In Mwanza region in the north-western part of the country, one study found a much lower prevalence; only 3.6% [7].

Scholars refer to the condition that the patients suffer from as “long uvula”, “diseased uvula” [32], or *kimeo* [33], the Swahili term for the local illness for which uvulectomy is the perceived appropriate treatment. In addition to “prolonged cough”, the reported symptoms of the condition include “fever, pain/difficulty in swallowing, difficulty in breathing and vomiting” [32]. In the medical literature, a swollen or elongated uvula is central in the clinical description of uvulitis [34].

A recent review of the medical records of more than 5000 paediatric patients who were received at the Emergency Department at the Muhimbili National Hospital in Dar es Salaam found that 1% of the admissions were related to recent uvulectomy. Close to half of them were diagnosed with pneumonia at the hospital [2]. However, the study did not have information on whether the caregivers had sought biomedical help before taking their child for cutting or not. In this cohort of 56 patients, 21% died, in contrast to 9% of the total sample. The authors conclude that “uvulectomy was associated with significant morbidity and mortality”, and argue that health campaigns should be carried out to reduce the prevalence [2].

Several scholars hold that traditional uvulectomy is illegal in Tanzania [32, 35], but uvulectomy is not mentioned in the Traditional and Alternative Medicines Act of 2002 [36], nor is it targeted as a harmful traditional practice by health authorities.

### The contribution of this study

Uvulectomy by folk practitioners is a relatively common form of folk health practice in Sub-Saharan Africa, and there is a need for more knowledge about the cultural ideas and practices that link by internal cultural logic to this form of surgery [5]. There is a small, but growing literature on uvulectomy in Tanzania. The studies published over the last 15 years are quantitative and have focused on prevalence and symptoms [2, 7, 8, 32]. Missing from the literature is caregivers’ perceptions of the folk illness *kimeo*, pathways of care, and experiences before and after taking a child for uvulectomy. To the best of our knowledge, this is the first work to focus on these aspects. Moreover, apart from a study from 1983 which included observations of one *kimeo* cutter in Dar es Salaam [31], and a survey of healers in a Congolese refugee camp in North-Western Tanzania and their perceptions of uvulectomy [25], the perspectives of the practitioners are missing from the literature. This study seeks to fill this research gap by addressing the following research questions: (1) How do parent caregivers in Dar es Salaam and the *kimeo* cutters perceive the folk illness *kimeo* and the efficacy of uvulectomy? (2) What factors enhance the social acceptability of uvulectomy to caregivers?

## Methods

### Setting of the study

The study was conducted in Dar es Salaam, largest Tanzanian city and the business hub of the country. The city is home to approximately 5.4 million people [37]. While many identify as urbanites, people also commonly identify with a rural homeland and one or more of the approximately 120 ethnic groups in the country. The United Republic of Tanzania does not publish statistics on religious affiliation. The coastal area, where Dar es Salaam is located, has a predominantly Muslim population, but the city itself is very heterogenous. People of different ethnic and religious backgrounds live in mixed neighbourhoods and are united by the national language Swahili. The relations between different ethnic and religious groups are overall amicable. Since formal jobs are scarce, a large proportion of the adult population are self-employed in the informal sector. Health care for children under five is free at public facilities but the quality of the services is often inadequate, and many people, including the poor, opt for private health services. In addition to professional health providers, Tanzanians make use of a wide range of healing paradigms [38–40].

### Study participants

Subjects of this study were children’s caregivers and folk practitioners of uvulectomy in Dar es Salaam. Caregivers here were primarily children’s parents; however, seven of the 43 caregivers were grandparents. Grandparents shared experiences from illness episodes both of their children and grandchildren. We interviewed both female and male caregivers, representing different locations within the city, ethnicity, gender, age, occupation, religion and levels of education. Our *kimeo* practitioner key consultants were four Muslim males. They live and work in three of the city’s five administrative districts, and within three different levels of urbanisation.

### Study design

The study had an ethnographic design with the main aim to understand the subjective realities of the participants and to get an insiders’ perspective of the folk illness *kimeo* and uvulectomy, in other words the “locally valid representations of illness” [41]. In medical anthropology, the term *illness* refers to the patients’ experience of their condition, in contrast to the term *disease*, which refers to professional health providers’ understanding of a given medical condition [9].

The study included ten focus group discussions (FGDs) [42–44] with caregivers and individual in-depth interviews (IDIs) [45, 46] with four *kimeo* cutters. The IDIs and FGDs were conducted by the two authors. We both took part in introducing the study to the participants and asking the introductory questions and follow-up questions. When we introduced ourselves to the participants, we explained that we were health researchers and that we had heard about the folk illness *kimeo* in another part of the country where we had worked previously, but that we did not fully understand this illness and thus wanted to learn more. We took care to communicate that we were unbiased and emphasised that we were interested in the participants’ own ideas and experiences [41]. None of the people we invited to participate refused, and none dropped out.

Both the FGDs and individual interviews were carried out solely in Swahili. This facilitated an atmosphere where the conversations and discussion could have a natural flow since there were no interruptions for translation purposes. In addition to notes during the interviews and FGDs, the first author took ethnographic field notes throughout the study [45].

*FGDs with caregivers* We used purposive sampling to recruit participants to the FGDs with the aim of obtaining variation with respect to geographical location within the city, gender, age, occupation, and level of education. The aim was to detect whether there were any differences in the perceptions of *kimeo* between these social groups. Because the public health authorities disapprove of uvulectomy, we decided that recruiting participants who know each other well would enhance trust and openness [32, 35]. We recruited participants who were either neighbours or members of a local association. Seven of the ten groups were recruited through our personal networks with the help of a gate keeper who asked his/her friends to participate in the study. The inclusion criteria were that the participants should have one or more children (of any age), know the other participants in the group, and be of the same sex as the gate keeper who was recruiting them. There were no exclusion criteria. The FDGs took place at one of the group members’ homes (in most cases a backyard or a veranda). The three other groups were mutual support associations whom we approached face to face and asked for their participation. The participants of these FDGs were self-employed and the FDGs were held at their place of work at a time that they found convenient. No members were excluded, since they all had children, and the groups were single sex, thus fulfilling the inclusion criteria. Apart from some young children who were with their mothers, no outsiders were present during the FDGs.

We purposefully recruited people who were part of networks because we believed this would facilitate openness and trust. In rotating saving clubs, women entrust each other with their savings and they meet regularly. In mutual support associations, a group of self-employed people, usually within the same sector, agree to support each other in times of extreme financial stress (e.g. the death of a family member).

We believe that by recruiting members to FDGs who knew each other well, and who trusted each other, we avoided some of the problems that other researchers who have carried out FGDs in Tanzania have encountered [44]. At the same time, there is the possibility that some participants would have been more open with strangers. Upon recruitment, we had no information about the participants’ experience with, or perceptions of, uvulectomy.

*Caregivers’ characteristics* Ten FGDs were carried out in three of the five administrative districts of Dar es Salaam: Ilala, Kinondoni, and Temeke. We recruited groups that were already established, so the number of participants varied, with the majority of the groups having four or five members (see Tables 1, 2). In each FGD, the participants were of the same sex, and were relatively equal in age and educational level. All the groups were ethnically mixed, but in two the participants hailed from the same region. The majority of the groups had both Christian and Muslim participants, but there were more Muslim participants compared to Christian. FDGs with males included members of three different mutual support associations (tailors, water sellers and taxi drivers), as well as two FDGs with neighbours (who had different occupations, mainly self-employed, but also some working in, or retired from, the formal sector). FDGs with women included self-employed members of saving clubs, as well as neighbours (two groups with home makers, one group with women who had retired from formal sector jobs).

**Table 1:** Focus groups discussion with female caregivers (*n* = 22)

Group	Occupation	Type of network	Age group	Education	Number of participants
01	Self-employed	Saving club	25–35	Primary	5
02	Self-employed	Saving club	25–35	Primary	5
03	Homemakers	Neighbours	25–35	Primary	4
04	Homemakers	Neighbours	35–45	Primary	5
05	Retired from formal sector jobs	Neighbours	60–70	Secondary and tertiary	3

**Table 2:** Focus group discussion with male caregivers (*n* = 21)

Group	Occupation	Type of network	Age group	Education	Number of participants
06	Self-employed	Mutual support association	25–45	Primary	4
07	Self-employed	Mutual support association	25–45	Primary and secondary	5
08	Self-employed	Mutual support association	25–45	Primary and secondary	4
09	Self-employed and formal sector jobs	Neighbours	25–45	Primary, secondary and tertiary	4
10	Retired from formal sector jobs	Neighbours	60–70	Secondary and tertiary	4

The FGDs were conducted in January 2012. We used an interview guide with open ended questions, focusing on perceptions of *kimeo* and uvulectomy. Many of the participants shared their own accounts of illness experiences, and this was often followed by a lively discussion. The FGDs lasted between 1 and 1.5 h, and the atmosphere was open and relaxed. The FDGs were recorded, and the authors took notes during and after the FGDs.

*IDIs and observations with practitioners* Four *kimeo* practitioners were purposely selected to cover different parts of the city: One in the city centre, two in two different residential areas of larger Dar es Salaam, and one in a semi-urban area at the outskirts of the Dar es Salaam region (see Table 3). The interviews took place at their clinics.

**Table 3:** Interviews with folk practitioners (*n* = 4)

	Pseudonym	Age group	Location	Services
1	Rashidi	50–60	City center	Uvulectomy Circumcision (males only) Herbal treatments for several illnesses, including asthma, peptic ulcer and cough that persists after uvulectomy
2	Khasim	40–50	Densely populated unplanned Area	Uvulectomy Herbal treatment for several illnesses
3	Omari	30–40	Densely populated unplanned Area	Uvulectomy Herbal treatment for several illnesses, including epigastric pain
4	Abasi	25–35	Semi-urban area (District 4)	Uvulectomy Herbal treatment for several illnesses

The first practitioner whom we asked for an interview said that he did not want to be recorded. This was probably because of the unclear legality of uvulectomy in Tanzania. Rapid notetaking was therefore used during the interviews with all four participants, and quotes from these interviews are therefore not verbatim in the strict sense. In addition to interviews, we observed the practitioners’ interaction with their clients before and after our interviews and took ethnographic notes.

The interviews were conducted in October 2011 and January 2012 and lasted between one and two hours. The interview guide was open ended and focused on the practitioners’ backgrounds, their perceptions of *kimeo*, their experiences with carrying out uvulectomies, their relationship to the authorities, and their perceptions of the professional health system. In December 2017, we conducted follow-up interviews with two of the practitioners. The main aim was to learn whether there were any changes in their practice and the way that the health authorities related to them. We also wanted to see the actual surgery and observed two cases of uvulectomy: a girl aged approximately five years, and a man in his mid-twenties.

*Kimeo cutters’ characteristics* The four *kimeo* cutters were between 25 and 65 years old. Two of them grew up in Dar es Salaam, while the two others grew up in rural areas and migrated to Dar es Salaam as adults. Three of them had completed primary school, while one had four years of secondary education. All four practitioners worked full time in their clinics and offered other services in addition to uvulectomy, like male circumcision and herbal treatments (see Table 3).

The clinics varied in appearance, but the structures looked simpler than clinics offering professional health services and three of them consisted of one room only and an outdoor waiting area. None of the clinics had paraphernalia commonly associated with traditional healers in Tanzania (whisks, calabashes, or textiles in the ritual colours red, white and black), nor offered divination, a service that is typically associated with Tanzanian healers. Three of the participants dressed in “Western” regular clothes: trousers and shirts. The fourth practitioner dressed in a stark white uniform, very similar to the uniforms worn by higher ranking health personnel at formal health institutions in Tanzania.

The four practitioners share some important characterises in common: they are male, they are Muslim, their ethnic background is from the Eastern part of Tanzania, and they are members of *Chama cha Utabibu Asilia* (The Association for Traditional Therapies). All four have learned their skill in apprenticeship with their father or older brother and carry out their practice from permanent clinics, which clients often refer to as *hospitali* (clinic, small hospital). Two of them are third generation *kimeo* specialists and operate clinics that have existed since the late colonial era. The two other clinics have existed for approximately five and twenty years respectively.

### Data analysis

Throughout the data collection period, the authors discussed the findings after each FDG and IDI. The second author transcribed verbatim the recorded FGDs and translated them to English. The two authors compared their hand-written notes from the IDIs and agreed on one set of typed notes for each practitioner, which included ethnographic observations.

The first author then read all transcripts and notes multiple times for familiarisation and coded the transcripts manually. She manually designed two coding trees referencing the coded information from the FDGs and IDIs. These coding trees reflected the ethnographic design of the study, which aimed at understanding care givers' and practitioners' perceptions and experiences with *kimeo* and uvulectomy, and the theoretical focus on the social acceptability of uvulectomy. The first author sorted data using the two coding trees to split data into related hierarchical conceptual themes [47]. Two main themes in the coding tree for the FDGs with caregivers were predefined based on the research questions (perceived causes of *kimeo* and pathways of care), while two new themes (trust in the practitioners and communication between caregivers and professional health workers) and one sub-theme (caregivers’ own experiences with going through uvulectomy) were identified during the analysis of the data. Likewise, the two main themes in the coding tree for the IDIs with the healers were predefined (perceived causes of *kimeo* and strategies to win the trust of caregivers and patients), while sub-themes were identified during the analysis process (procedures of the surgery and membership in associations). Acknowledging that illness representations are often varied within a community [41], we looked both for perceptions of *kimeo* and uvulectomy that many of the participants shared, as well as perceptions that only one or two of the participants voiced (outliers in the data).

## Results

This section will first present the findings from the interviews with the *kimeo* cutters before we turn to caregivers and their perceptions of *kimeo* and their experiences with uvulectomy by folk practitioners.

### Practitioners

#### Kimeo symptoms and perceived causes

All of the four *kimeo* cutters mentioned *homa* (fever, general weakness/illness) and a persistent cough, often causing lack of sleep, as the main symptoms of *kimeo*. All four diagnose their patients by observing the uvula and asking patients for symptoms. The practitioners described *kimeo* as a swollen and elongated uvula. In the early phase of *kimeo*, the uvula looks like normal skin, but then it “ripens”, and it may become very difficult to eat. This, in their view, is one of the reasons why both adults and children often lose weight when they suffer from *kimeo*.

Two of the practitioners associated *kimeo* with hot weather. Abasi explained that he associates *kimeo* with a dry cough, in contrast to the productive cough that comes with cold weather. He concluded that this is the reason why *kimeo* is most common in hot places, like the Eastern part of the country and along the coast. Three of the practitioners said that a child cannot be born with *kimeo*, while Omari said that some children are born with *kimeo* because their mothers fail to get nutritious food when they are pregnant, and that it could also be caused by a child's DNA.

Three of the *kimeo* cutters associated *kimeo* with tuberculosis. Abasi, for example, said that most of his patients have tried all the medicine that hospitals can offer, without getting cured, and that living with *kimeo* by delaying cutting can be dangerous by leading to additional illnesses: “I have cut many people who are late, and then they look like they have some kind of TB.” Khasim explained that when the uvula swells, it contains microorganisms (*wadudu*):

Some of those microorganisms will spread and cause illness and some will die. So the problem starts there, and then some can get TB (…). They are unable to spit out (the microorganisms) once it bursts.

Rashidi, when asked how he differentiates between *kimeo* and tuberculosis, responded in this way: 

"Some people run away from the hospital, then we tell them to go back. And some have *kimeo*, but also other illnesses. Then, we also tell them to go to the hospital."

*Kimeo* cutters (and other Tanzanians) often use the expression “other illnesses” about HIV/AIDS, and tuberculosis is relatively common among HIV + patients. None of the practitioners mentioned supernatural or personalistic causes of *kimeo*.

*The cutting* All four practitioners have several sets of metal forceps which they use for cutting their patients’ uvulas, and they sterilise the equipment in boiling water with herbs. Three reported performing between 10 and 15 uvulectomies per day, while one did only two on average. Three charged TZS 10,000 (approximately USD 4.3), while the fourth, who operates in a semi-rural area, charged less, TZS 7000 (approximately USD 3). After the cutting, the patients gargle water with herbs to help stop the bleeding and to clean the wound. One of the practitioners reported that he prescribes antibiotics (amoxicillin) for adult patients, to be taken for a week after the uvulectomy.

There were some differences between the four concerning what time of the day the cutting could be carried out. Abasi said that he never carries out the cutting after 10 am, because “after that, the blood is more active, and the cutting can lead to more bleeding”. The other three did not have such restrictions. In the words of Omari: “Many think that the sun makes the body boil, but due to modern instruments, it is no longer important to do it in the morning”. Khasim had yet another explanation for why the procedure was formerly always done in the morning. He argued that in the old times, when the practitioners used a thread, razor blade, and a wooden spoon, the clients often started vomiting, and the cutting was therefore done in the morning, and clients were told not to eat beforehand. With the use of modern forceps, he explained, the procedure is much quicker, and it is no longer a problem that the clients have eaten beforehand.

We observed that the surgery is done quickly: it takes three to four seconds only. The practitioner uses one pair of forceps to hold the uvula, and another pair to cut it. There is no cauterisation nor stitches on the wound. A male patient, approximately 25 years old, did not show any signs of pain. A female patient, approximately 5 years old, who sat on her mother’s lap during the cutting, whined for a few minutes after the cutting. In both cases, the *kimeo* specialist wrapped the amputated uvula in paper and gave it to the patient/caregiver.

We asked the *kimeo* cutters whether the cutting can be dangerous or whether any of their patients have died. All four denied that this had ever happened. However, two of them admitted that some patients can start bleeding after the cutting: “If we encounter heavy bleeding, we give the patients some medicine and it stops”. Rashidi, the most senior of the four *kimeo* cutters, disclosed that in very rare instances, the patient continues to bleed heavily, and they then tell them to go to hospital. He added that at hospitals, some doctors treat such patients, while others tell them to go back to the *kimeo* cutter.

#### Winning patients’ and caregivers’ trust

The practitioners emphasised different aspects when talking about how they gain patients' and caregivers’ trust. Rashidi talked about technical expertise and said that one has to watch the procedure for at least two to three years before one can carry it out; adding that it is much harder to learn than driving a car. Omari said that it is important to attend people in a nice way. He argued that health workers in the professional health system often fail to do this:

Medical people do not show love to the patients. Sometimes they just write, without even looking at the patient’s face. What is needed is words, advice, and a good relationship.

For Abasi, not coming through as greedy was very important for gaining patients’ trust. He explained that his father had considered his children's personality carefully when deciding whom among his eight children should work with him in an apprenticeship. He wanted someone calm, and who was not greedy, as one should not be tempted to cut people who do not suffer from *kimeo*. He emphasised that he adheres to what his father taught him about not being greedy:

There were many of those who came here this morning whom I told that they don’t have it. I said: “If you go somewhere else (to be cut) you will suffer for no reason, and then you will start to get problems.” (…) I explained to them: “Go to a hospital and get tested, you will get some other kind of treatment”.

Although there was some variation between the practitioners in what they emphasised as enhancing trust among their clients, all of them appeared to be conscious about this aspect of their work.

*Licenses to practice and confidence in own expertise* All the practitioners were aware of the fact that Tanzanian health authorities are ambivalent or sceptical towards the practice of uvulectomy and only one of the four clinics had a signpost outside. All four were members of *Chama cha Utabibu Asilia* (The Association for Traditional Therapies), which provides licenses to their members in line with the law that regulates Traditional, Complementary, and Alternative medicine (TCMA) in the country. Two of them carried their member cards around their necks, and on the cards, they were entitled *mganga*, a Swahili word that is used for both medical doctors and healers. Two of the providers specifically referred to the license as a form of approval from the government. In the words of one of them:

We have the license, and we have been here for so many years – my father before me - so more than 50 years! The government people tell us just to go ahead. Sometimes a police officer comes here, and I’m thinking to myself – ‘I will be arrested today’ – but then it’s just a patient!

The licenses that come with the membership in the association give the providers a sense of legality, although they know that the health authorities disagree with the form of surgery that they carry out. Omari emphasised that they use the same equipment as in hospitals and that many come to him after having been treated in the professional health system without getting well:

Medical people do not explain *kimeo* properly, they say it is not a disease. They say that the uvula acts like a police force to safeguard the body. But there are countries where the military force can harm the citizens, right?

All the providers appeared to be confident that *kimeo* is an illness that modern, professional medicine simply has not yet understood. This perception was shared by the great majority of the caregivers, to whom we now turn.

### Caregivers

The 43 caregivers who took part in this study reported to have taken all together 18 children for cutting and six of the participants had memories of having gone through uvulectomy themselves.

#### Perceptions of kimeo

The analysis showed that caregivers share coherent ethnomedical understanding of *kimeo*. We did not find any gendered differences in the way that participants talked about *kimeo* and uvulectomy, nor any systematic differences between the different districts of the city. Muslim caregivers who originated in Eastern Tanzania knew more about *kimeo* than Christian caregivers from Northern Tanzania, but we did not systematically look into differences between different ethnic and religious groups.

*Belief in kimeo* All participants in the FGDs knew of *kimeo* and uvulectomy and the great majority personally knew someone who had suffered from *kimeo*. The overall sentiment across all focus groups was that *kimeo* exists and is a threat to children’s health. Only one participant among the 43, a Christian woman in her 30 s, said that she did not believe in the existence of *kimeo*. Two other participants, both male and Christian, explained that they had initially been sceptical about the existence of *kimeo*, but that they had changed their mind after having witnessed children in their neighbourhood getting well from prolonged illness after they had been cut.

All participants agreed that *kimeo* does not belong to the sphere of tradition, ritual, or religion (*mila na desturi, dini*), but several participants who were immigrants to the city mentioned that *kimeo* is not common in their home area and that they had only heard about it when they came to Dar es Salaam.

*Kimeo symptoms and severity of the condition* In all FGDs, participants described *kimeo* as a growth, elongation, or swelling of the uvula, and they mentioned cough as the main symptom which in young children is commonly accompanied by vomiting. In the words of one of the elderly female participants who had retired from a formal sector job:

The child coughs a lot, you give him cough mixtures, but he doesn't get cured. If it's a young child, he gets a high fever in the evenings, he coughs, and when you breastfeed, the child vomits, and he also vomits the food you give him. He becomes weak because nothing stays in his stomach. (FGD 05)

While cough and vomiting were the most mentioned symptoms of *kimeo*, some participants also mentioned that an elongated uvula could lead to difficulties in breathing. Vomiting and difficulties to eat were described as symptoms that made affected children very weak, and difficulty to breathe was regarded as a very serious symptom. Several participants emphasised that the illness can be life-threatening if uvulectomy is not performed and this was linked to the importance of not delaying taking the child for uvulectomy:

The uvula elongates. If it increases and swells - they say that if it continues to swell and then bursts - at the end of the day that puss will make you die. It is therefore extremely important to hurry up and cut it early. (FGD 06)

Participants also said that the *kimeo* cutter must make sure to catch the amputated part to avoid it being swallowed by the patient, since it contains the dangerous puss that can then spread to the body. In three of the ten groups, one or more participants associated this puss with tuberculosis which would eventually lead to the patient’s death. While there was overall consensus that if left untreated, *kimeo* is dangerous and potentially life-threatening, only one participant reported that he knew of a child who had died due to delayed cutting.

#### Trust in the practitioners

Participants mentioned six different *kimeo* specialists in Dar es Salaam and several referred to them by name. The great majority of the participants referred to the place where the uvulectomy is performed as a hospital/clinic (*hospitali*) and the folk practitioner as “*kimeo* cutter” (*mkata kimeo),* or simply “expert” (*mtaalam*). Some referred to the *kimeo* specialist as “doctor” (*daktari*). No participants referred to them with the term healer/traditional doctor (*mganga wa kienyeji*).

Several participants emphasised that the *kimeo* cutters are experts on what they do and that it takes "great expertise (*utaalamu mkubwa*) to take a scissor down the throat”. The fact that the folk practitioners use the same equipment as hospital staff enhanced trust:

You see their equipment – it is the same as they have at hospitals. And they have identity cards. (FGD 07)

Another factor that enhanced trust was the fact that *kimeo* cutters turn some patients away. This was seen as a poof that they are not greedy:

If it is true that the child has it, they tell you, but if the child doesn’t have it, they tell the mother: “Mama, take your child back home, there is no *kimeo*”. Those people are not greedy for money. (FGD 01)

Caregivers then emphasised the same issues that the practitioners themselves mentioned as important for building trust: licenses, professionalism, and the fact that they do not cut all clients but turn some of them away.

#### Pathways of care for children

Approximately one third of the participants reported that they had taken one or more of their children for uvulectomy. The great majority said that they had taken their child to a professional health provider as a first recourse. A middle-aged man explained that when his son was four months old, he had a terrible cough:

They diagnosed him with malaria and cough at the hospital. He was given cough mixtures and he completed the prescribed doses (…), but he didn’t get well. Then people said: “It is *homa homa* (fever, general weakness/illness), he has *kimeo*”, so they said he should be cut. I agreed, and truly, he became well after they took away that *kimeo*. (…) You will go and get treatment for that *homa* until you have used all kinds of medications, but then (after cutting), just one day and the child is well! (FGD 06)

Many participants told similar stories—if care seeking through the professional health system failed to improve the child’s condition, they had listened to advice from others that they should take their child for cutting. It is also notable that although most participants referred to the illness period as lasting for a long time, it became clear that in some of the cases, the illness had lasted for a week only, but they felt that in that period, their child suffered badly.

*Bypassing professional health care* A few participants explained that based on their experience with *kimeo* or advice from relatives or friends, they had taken their child directly for cutting, without accessing professional health care first. One woman reported that her first child suffered from a severe cough when he was nine months old. He had started losing weight due to the coughing and vomiting and she took him to several private clinics that offer professional services, but there was no improvement. He got well only after she had taken him for cutting. When her second child, who was then five months old, started coughing, she took him directly to a *kimeo* specialist:

From the experience I had with my first born (…) I myself decided that this must be *kimeo*. And when I took him to the place where they remove it, they confirmed that he had *kimeo*. (FDG02)

For some caregivers then, earlier personal experience guided their decision to bypass professional health care for their children.

*Own experiences with going through uvulectomy influence care seeking* Six of the 43 participants remembered going through uvulectomy themselves and all reported that for them, the cutting had given relief to their symptoms. A tailor in his early 40 s explained that he had uvulectomy performed when he was 23. He had been coughing for approximately a month and was given cough mixtures, but he did not get better. Three days after the cutting he got well and the condition has not come back. When his daughter got sick when she was three years old, he decided to take her for cutting as well. She was coughing a lot and they could see that the uvula was very long, touching her throat. After the cutting, she reportedly never suffered from a prolonged cough again. Another male participant, a taxi driver, was around 14 years old when he was cut. He was coughing a lot and was “sleeping the whole afternoon”. He went for professional health care but did not get better until he had gone through uvulectomy. Like the tailor, the taxi driver chose to take his own children for cutting. His twins were then two years old. For these two participants then, their own positive experiences with uvulectomy were a central factor influencing their decision to take their children for cutting.

#### Communication between caregivers and formal health workers

In all groups, there was agreement that health authorities disapprove of uvulectomy and claim that a disease like *kimeo* does not exist. One male caregiver argued that since the professional health workers do not know the illness, they are unable to treat *kimeo*:

The normal hospitals (*hospitali za kwaida*) aren’t aware of it, hence they can’t treat it.

When asked whether they discussed *kimeo* with health workers, most caregivers said no. One mother explained how this made her keep silent:

The doctor gives you medication to prevent the child from vomiting, but it just continues. When you try to tell the doctor that there are other treatments, he will tell you that you shouldn’t teach him how to do his work. (…) Then you decide to keep quiet. What else can you do? (FGD02)

As mentioned earlier, only one of the 43 participants said that she did not believe in the existence of *kimeo*. After listening to another participant’s narration about taking her child for uvulectomy, she said:

I almost did the same thing, but when I took my baby who had similar symptoms to the doctor, he insisted that I shouldn’t take him for uvula cutting. He told me about a mother who lost her child after trying to hide that she had taken the baby for cutting. The baby died because of losing a lot of blood. (FGD04)

This woman recounted that her child’s uvula had become very long, but after three days on the prescribed medication the child got well and did not vomit anymore. She had taken her child to a private clinic, and the medication cost her TZS 18,000 (approximately USD 7.8). This is almost twice as much as *kimeo* specialists charge for cutting.

## Discussion

Our study findings indicate that uvulectomy is a relatively common treatment for children in Dar es Salaam. This shows that the predictions in the 1960s and 1980s, suggesting that “traditional uvulectomy” would be abolished, did not hold true [22, 30]. We have identified four factors that contribute to the social acceptability of uvulectomy in Dar es Salaam: (1) Caregivers perceive that children who suffer from cough and vomiting do not get well after visiting professional health providers (2) Caregivers believe that professional health providers are unable to treat the folk illness *kimeo,* and some believe that *kimeo* is an acute, life-threatening condition; (3) Caregivers have experienced that children who suffer from *kimeo* get well after uvula amputation, and they share their positive experiences with others; (4) Caregivers trust the folk health providers who carry out the cutting as experts in treating *kimeo.* In the following, we will discuss each of these factors.

### Children do not get well after visits to professional health providers

It has been argued that the use of folk medicine is closely associated with distance to formal health care [1]. We conducted our study in Tanzania’s largest city, where the distance to formal health care is not a limiting factor, and where health services for children under 5 are free at public facilities. The majority of our participants who reported that they had taken a child for cutting said that they had first brought their child to a professional health provider. There is reason to believe that some of the children who are taken for uvulectomies have received inadequate care at formal facilities, either in terms of diagnosis, the quality of the prescribed medication, or both. Several studies have shown that children are not examined well enough at public clinics in Tanzania [48, 49] and one study of child morbidity in rural Tanzania found that in nearly 80% of malaria-attributable deaths, caregivers had used professional care as the first resort [33].

Caregivers describe the typical symptoms of *kimeo* as cough, fever, vomiting, weakness, fever, and difficulties in breathing. These symptoms can occur with a range of different clinical conditions, including malaria and pneumonia. Close to half of the children who had gone through uvulectomy and who had been admitted to the emergency department at Muhimibili national hospital in Dar es Salaam, were found to have pneumonia and/or malaria, while one in five had HIV and/or severe anaemia and malnutrition [2]. Since the studied medical records do not have information about care seeking prior to the uvulectomy, we do not know whether these children had been taken to a professional provider prior to the cutting or not. It is also important to note that this sample from the emergency department is not representative of children who have been taken for cutting. There is reason to believe that many of the children who are taken for uvulectomy, and who do not end up at the emergency department but get well, suffered from conditions that were self-limiting [30]. None of the participants in our study had experienced negative outcomes from the cutting itself, but the *kimeo* practitioners said that bleeding happens occasionally. The hospital-based study cited above found Upper GI bleeding in 46% of the children and septicaemia in 6%.

Tanzania has a high burden of tuberculosis [50]. In the 1960s, a study on uvulectomy in Dar es Salaam found that many of the adult patients who had gone through the cutting suffered from tuberculosis [30]. Two of the four practitioners and some of the participants in this study associated *kimeo* with tuberculosis, but tuberculosis was not among the diagnoses in the hospital based study cited above [2]. This can indicate that tuberculosis is not a common underlying illness in children who are taken for cutting, or that it is hard to diagnose tuberculosis in young children [51].

### Professional health providers are unable to treat the folk illness kimeo and kimeo is potentially life-threatening

The great majority of the caregivers who participated in this study believed that professional health providers are incapable of treating a swollen or elongated uvula. Some of our participants firmly believed that a child suffering from *kimeo* will die if uvulectomy is not performed, or if the cutting is delayed since the elongated uvula would then rupture and cause the child's death. While this belief has been reported also from Ethiopia and Hausa-speaking communities in Niger and Nigeria [4, 23, 24], it has not previously been reported in studies from Tanzania [2, 30, 32]. We argue that this fear may be an important factor for the social acceptability of uvulectomy in Dar es Salaam and the sense of urgency that some caregivers feel.

### The perceived efficacy of uvulectomy and few negative outcomes

Many of the participants reported that they had witnessed that the cutting is a very efficient way to get well from *kimeo*. The population in Dar es Salaam is mixed in terms of ethnic composition and religious affiliation, and although we recruited participants to reflect this diversity, we did not attempt to systematically investigate differences in perceptions between different groups. However, we did find that participants who hailed from areas in Tanzania where *kimeo* is unknown, and who had initially been sceptical to this local illness, had changed their mind after witnessing children get well after having been cut. A central finding of this study is that caregivers rely on their personal experiences, or advice from family and friends when choosing to take a child for uvulectomy. Medical anthropologists have pointed out that experiences “are generated relationally” [52] and other studies from Tanzania have shown that social relations are central for treatment-seeking practices, and that trust in certain forms of treatment is “reinforced through social recommendation” [40]. There is therefore a possibility that the acceptability of uvulectomy can increase in the urban context, rather than decrease.

Only one of the 43 caregivers that participated in the FGDs mentioned potential negative effects of uvulectomy and none of the participants had personal experiences with negative outcomes. The practitioners admitted that excessive bleeding did happen, but very seldom. This is consistent with an earlier study which found that that “serious after-effects can occur after uvulectomy, but are probably not very common” [30].

### Trust in informal health providers’ professionalism

There has been a tendency in the public health literature to categorise professional health care and folk health care as two separate spheres. However, if we are to understand caregivers’ decision-making, we need to accept that for many of them, the distinction between professional and folk health care is blurry. Our results show that caregivers to a large extent refer to the places where the uvulectomies are performed with the same word as they use for the places which offer professional health care: *hospitali* (clinic/hospital). Previous ethnographic studies of healing in Tanzania similarly found that the “traditional” and “modern” are inseparable [38, 39].

We found that trust in the practitioners is partly based on their hybrid practices—that is, their use of modern medical equipment, the fact that they sterilise the equipment that they use, and their technical skills in performing the surgery quickly and efficiently, causing limited pain—and a quick relief from their symptoms for many of the patients.

Like folk practitioners in other contexts, the *kimeo* cutters borrow from the prestige of biomedicine [11, 20, 21] “to legitimate themselves” [53]. They do this by selectively adopting and modifying elements of biomedical care [54], like using forceps and dressing in a stark white uniform, thus employing “self-made medical authority” [55]. In addition to legitimising their services by employing hybrid practices, the *kimeo* specialists make their services attractive to their clients by, for example, taking care to listen to them well, and by not coming across as greedy. For some caregivers, the fact that the practitioners turn away some of the patients was seen as a sign of quality of care and professionalism. Others referred to the fact that the *kimeo* cutters carry identity cards as a factor enhancing their trust in them.

In West Africa, uvulectomy is associated with Muslim communities and often carried out by male barbers [24]. The four *kimeo* cutters whom we interviewed were male and Muslim. However, the caregivers (who included Christians) denied that *kimeo* or uvulectomy had anything to do with religion. It is also worth noting that during our research in Morogoro region in Tanzania, we met a female, Christian *kimeo* cutter.

### Policy implications and the need for further research

Worldwide, the legal status of folk practitioners’ health services is often unclear [53]. Tanzanian authorities have an ambivalent relationship to folk health services [19]. On the one hand, biomedical clinicians and government campaigns encourage citizens to use modern and formal health care, people who use folk medicine, particularly the services of traditional birth assistants, tend to be labelled as backwards in public discourse, and there is a “government stance on discouraging dangerous traditional practices” [2]. On the other hand, WHO has signalled that what they have labelled “Traditional, Complementary, and Alternative Medicine” (TCAM) can be a useful supplement to improve public health [56]. Tanzanian health authorities have adopted this term, and the law that regulates TCMA in the country, the Traditional and Alternative Medicines Act of 2002, permits not only for controlling and regulating TCMA, but also for promoting TCMA [36]. Earlier research on uvulectomy in Dar es Salaam has suggested that most of the practitioners “are not recognised by the ministry of health” [2]. In interviews with us, the practitioners emphasised that they need to register with an organisation for traditional healers (*waganga wa kienyeji*) to get a licence, and they regarded their license as an official recognition of uvulectomy. To what degree the Ministry agrees with this interpretation is an open question.

One way to address the potentially negative outcomes of uvulectomy is for professional health workers to discuss uvulectomy openly with caregivers whose children suffer from the symptoms that are commonly associated with *kimeo*. Our findings show that at professional facilities, health workers and caregivers very seldom bring up the question of *kimeo* or uvulectomy*.*

Several authors have argued that health education is needed to abolish uvulectomy [2, 22, 30]. In Ethiopia, where uvulectomy is often done in the neonatal period, health education on the dangers of uvulectomy has been introduced during antenatal and postnatal visits, and neonates born to parents who have received such counselling were found to be much less likely to be taken for uvulectomy than other neonates [4].

Health campaigns have proved successful in changing Tanzanian caregivers’ health seeking behaviour for another local illness category, namely *degedege* (convulsions). A study in south-eastern Tanzania found that parents of children with *degedege* traditionally did not associate the convulsions with malaria, and commonly took children with this condition to healers. Health campaigns to “promote a new understanding” linking the local illness category *degedege* to malaria proved to change caregivers’ treatment-seeking behaviour [13]. A challenge in the case of *kimeo,* however, is that up to now, no studies have been carried out to establish what kind of clinical conditions children who are taken for cutting suffer from. Further research is therefore needed before designing efficient health campaigns. Such research should preferably be conducted in partnership with one or more of the associations for folk practitioners in Tanzania.

## Limitations

We interviewed four *kimeo* cutters who had some central perspectives in common, but who differed in the ways that they describe the aetiology of *kimeo*. Interviews with additional practitioners would have offered more insights to this variation, and participant observation over time, rather than interviews, would have given us a better understanding of *kimeo* cutters’ practices. However, budget constraints limited the present research. There is a dichotomy in Tanzania regarding the use of folk health services, where some see it as beneficial, while others strongly disapprove of it. We believe that introducing the study to the caregivers who participated in the focus groups in a non-judgemental way facilitated openness among the participants. However, some of the caregivers may still have overreported the use of professional providers. At the same time, the fact that in all the groups some of the participants were immediately very vocal about the existence of *kimeo*, could have hindered other participants to voice their scepticism.

## Conclusion

The folk illness *kimeo* is perceived as potentially life threatening. For the large majority of the caregivers who participated in this study, the only *kimeo* remedy is amputation of the uvula by a folk practitioner. Improved quality of care at professional clinics, including more culturally respectful, yet open communication about folk illnesses like *kimeo*, plus information about the potential dangers of uvulectomy during routine postnatal care, can potentially reduce morbidity and mortality. Danger exists from the child’s postponing of biomedical diagnosis of any underlying illnesses causing the child’s cough and uvulitis (and often vomiting, fever, and weakness). These underlying illnesses may themselves be symptoms of a larger systemic disease beyond, yet including, the *kimeo* symptoms in cases that are not isolated to or caused by the uvula. There is also potential danger of excess bleeding, gagging, vomiting, sepsis, and other negative side effects from the cutting. More research is needed to establish the clinical biomedical conditions that children suffer from when taken for uvulectomy. Increased focus on uvulectomy and the folk illness *kimeo* is particularly important during the covid-19 pandemic when more people are likely to experience cough and weakness.

## Data Availability

The datasets used/or analysed during the current study are available from the corresponding author on reasonable request.
